# Endoscopic Cystogastrostomy: Minimally Invasive Approach for Pancreatic Pseudocyst

**Published:** 2015-01-01

**Authors:** Gull-Zareen Khan Sial, Abid Quddus Qazi, Mohammed Aasim Yusuf

**Affiliations:** 1 Department of Paediatric Oncology, Shaukat Khanum Memorial Cancer Hospital and Research Centre, Lahore.; 2 Department of Surgery, Shaukat Khanum Memorial Cancer Hospital and Research Centre, Lahore.; 3 Department of Gastroenterology, Shaukat Khanum Memorial Cancer Hospital and Research Centre, Lahore.

**Keywords:** Pancreatic pseudocyst, Paediatric patients, Endoscopic cystogastrostomy

## Abstract

Pancreatic pseudocysts in children are not uncommon. Non-resolving pseudocysts often require surgical intervention. Endoscopic cystogastrostomy is a minimally invasive procedure which is recommended for this condition. We report a large pancreatic pseudocyst in a 4-year old child, which developed following therapy with PEG-Asparaginase for acute lymphoblastic leukemia. It was managed with minimally invasive procedure.

## INTRODUCTION

Pancreatic pseudocyst in children may be seen after pancreatic injury. Most pseudocysts resolve with supportive treatment. However in case of failure of expectant treatment, open cystogastrostomy has been a standard procedure.[1] With the advent of modern endoscopes and experience it is now possible, in appropriate settings, to treat these pseudocysts safely using minimal access techniques.

## CASE REPORT

A 4-year old male, being treated for acute lymphoblastic leukemia, developed acute pancreatitis after receiving PEG-Asparaginase during the induction phase of chemotherapy. There was symptomatic relief with medical management. However, three weeks later, the patient presented with fever, abdominal pain, vomiting and marked epigastric distension. Ultrasound of the abdomen revealed a large pancreatic pseudocyst (PP) measuring 85 x 70mm. It was initially managed conservatively by keeping the patient nil by mouth, on intravenous fluids, antibiotics and prophylactic fluconazole for 5 days. Due to persistent vomiting, he was started on elemental diet via a naso-jejunal tube, which he tolerated well. Re-evaluation ultrasound after 5 weeks of non-operative management revealed marginal increase in the size of the pseudocyst, which now measured 93 x 82mm. Therefore the patient was referred for endoscopic cystogastrostomy. Pre-procedure magnetic resonance cholangio-pancreatography (MRCP) showed a well-encapsulated but thin-walled cystic lesion in front of the body and tail of pancreas, immediately behind the stomach, with gross forward displacement of the stomach (Fig.1). The pancreatic head and neck appeared unremarkable. Some cystic changes and low signal intensities were seen in the region of the pancreatic tail. The common bile duct (CBD), extra and intra-hepatic biliary channels did not show any abnormality. For endoscopic ultrasound guided drainage, an Olympus linear echoendoscope was introduced via the mouth, under general anaesthesia and advanced to the stomach, from where the cyst was identified (Fig.2). Using a 19G needle (Fig.2), the cyst was punctured and a cystotome was then used to enlarge the track, over a guide-wire. Two 7 Fr 7cm silicon double pigtail stents were then inserted into the cyst. A sample of orange coloured fluid was retained for analysis. Amylase level in this fluid was subsequently reported as 14,254U/L. The patient was kept under follow-up with serial ultrasound scans for complete resolution/recurrence of pseudocyst. Following complete resolution, the stents were removed endoscopically after eight months and the child remained well.

**Figure:**
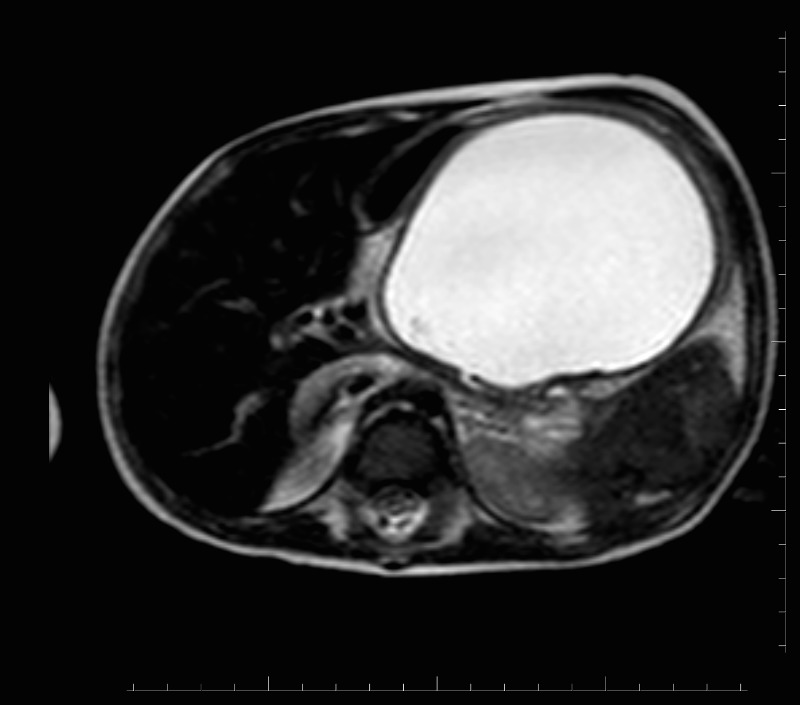
Figure 1: MRI showing a large cystic collection of fluid in front of body and tail of pancreas, pushing the stomach anteriorly.

**Figure:**
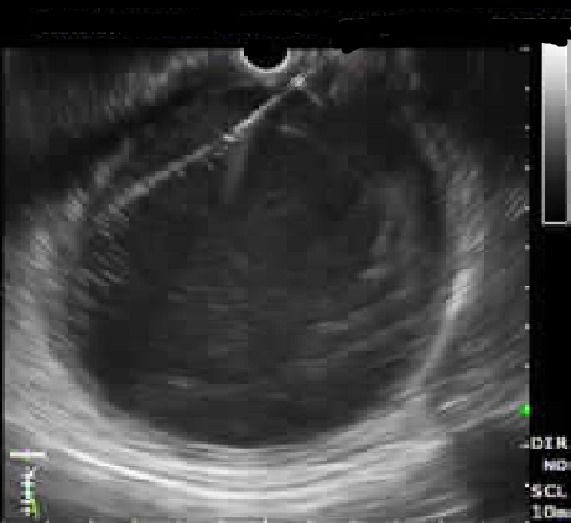
Figure 2: Showing endoscopic insertion of needle (arrow) in to the pseudocyst to aspirate the fluid and subsequently insert guide wire.

## DISCUSSION

Various treatment options are available for the management of PP, such as open surgery, per-cutaneous drainage, laparoscopic and endoscopic cystogastrostomy. Initially, most patients are given a trial of non-operative management by maintaining good hydration, analgesia, prophylactic antibiotic coverage and parenteral or enteral nutrition in the hope that the PP may undergo spontaneous resolution.[2,3] If its size is more than 5-6 cm and progressively increasing, as happened in our case, then the case for intervention becomes stronger.

Open cystogastrostomy (OCG) has been considered the gold standard for drainage of pseudocysts.[4] Percutaneous cystogastrostomy (PCG) is a less preferred technique due to high risk of recurrence and pancreatic fistula formation.[5] Laparoscopic cystogastrostomy (LCG) has been frequently used and has consistently produced good results. However in comparison to endoscopic cystogastrostomy, it is still a more extensive procedure.[6] Three to four ports are used to create cystogastrostomy of Roux-En-Y jejunostomy. Occasionally it may require conversion into an open procedure.[7] This procedure may be reserved for failed endoscopic procedures.

Endoscopic cystogastrostomy (ECG) is superior to the aforementioned techniques in being minimally invasive with quick post-procedure recovery and consequently shorter hospital stay. Our patient was discharged home after overnight post-procedure monitoring. The risk of recurrence of symptoms due to stent clogging or migration can be lessened by use of two-pigtail stents.[8] With the aid of endoscopic ultrasonography, the risk of haemorrhage due to trauma to local vessels is further minimized.[9] There is limited data available regarding ECG in children.[10] Although there is a risk of recurrence even with this procedure, using it as the first modality is the best choice due to the minimally invasive nature of the procedure. Endoscopic cyst-gastrostomy was found to be safe and well-tolerated procedure in index case and we recommend that it should be considered in the management of children with pancreatic pseudocysts.

## Footnotes

**Source of Support:** Nil

**Conflict of Interest:** None declared

